# Effect of Hydration and Carbonation Progress on the Porosity and Permeability of Cement Pastes

**DOI:** 10.3390/ma12010192

**Published:** 2019-01-08

**Authors:** Tomasz Tracz, Tomasz Zdeb

**Affiliations:** Faculty of Civil Engineering, Cracow University of Technology, Warszawska 24, 31-155 Cracow, Poland; tzdeb@pk.edu.pl

**Keywords:** cement paste, water-cement ratio, open porosity, helium porosity, MIP porosity, gas permeability, carbonation, hydration degree

## Abstract

This paper presents the results of comprehensive cement paste porosity and gas permeability tests. The tests conducted concerned ordinary Portland cement (OPC) cement pastes with varying water-cement ratios ranging from 0.3 to 0.6. The tests were conducted after the curing of cement paste for 90 days and two years under laboratory conditions. Open porosity was determined using three methods: helium pycnometry, mercury intrusion porosimetry, and water saturation. Permeability was determined using a modified RILEM-Cembureau method. The results obtained demonstrated that permeability does not change significantly over time despite the observed material shifts in open porosity characteristics caused both by further progress in hydration and by the carbonation process that occurs. The results of the tests conducted also permitted the quantitative determination of the impact of the water-cement ratio, age, and the progress of carbonation on open porosity measured using different methods and also on the gas permeability of the pastes.

## 1. Introduction

Currently the requirements concerning the durability of engineering structures are becoming ever more stringent. We expect structures to have the longest possible service life during which time no repair work should be required despite considerable environmental aggression being frequently present. In the literature, there is a constant search for diagnostic characteristics which could predict the resistance of materials exposed to specific environmental conditions as faithfully as possible. Many scientific and engineering communities are working intensely to develop adequate methods for assessing the potential durability of the materials used in the construction industry. Dynamically developing knowledge about the behavior of materials during their service life, insight into processes that compromise durability, and finally state-of-the-art research equipment offer the potential to provide considerable achievements in this area. Obviously, this does not mean that it is sufficient to replicate the technical solutions that have already been tested; on the contrary, this potential provides an additional incentive for researchers to develop their methods and offers new opportunities to gain knowledge.

### 1.1. Methods for Measuring the Permeability of Cementitious Composites

The most popular methods for assessing the ability of cementitious materials to transport liquid environmental media have been the measurement of water permeability and water absorption, which are described in many standards [1,2,3,4,5]. Since the new generation of cementitious materials emerged (high-performance concretes, reactive powder concretes, etc.) among whose main distinguishing characteristics are the significant reduction of both total porosity and its distribution, the popular water permeability test has become rather ineffective. Distinguishing between the degrees to which internal concrete structures are accessible to water has turned out to be very difficult or even impossible due to the tight texture of these materials [6]. Much subtler possibilities of distinguishing the degree to which internal material structures are accessible have been offered by measurements that use methods based on gas flow. Previously, such methods were used primarily for rock materials [7,8,9]. Concrete permeability determined in this manner reflects the degree of its accessibility to gases, which are present in the environment and are either themselves harmful to reinforcing steel (O_2_) or reduce the concrete’s capacity to protect reinforcing steel (CO_2_). Irrespective of this, in some applications (e.g., internal lining of underground geo-reservoirs for gas storage purposes), knowledge about the permeability of concrete to gaseous media is in fact required.

One of the most popular methods for assessing gas permeability is the laboratory-based RILEM-Cembureau method [10]. The gases most commonly used in this method are oxygen, nitrogen, or air. The advantage of using gases that do not react with the material’s skeleton in permeability tests is the possibility of repeating such tests many times without the potential effects of using water such as leaching water-soluble components out of the skeleton or advancing hydration. The value of the permeability coefficient k (m^2^) for stabilized gas flow is determined using Darcy’s law.

The literature review carried out indicates that the gas permeability tests conducted so far mainly concerned concrete as a whole, and hence the permeability measured could be termed global, since it illustrates the transport of gas molecules that takes place through a network of open pores contained in cement paste, in the interfacial transition zone and in the aggregate at the same time. Due to the fact that the permeability of concrete components may differ even by several orders of magnitude, this permeability should be determined separately for each individual component and the impact of different factors on its variability should be established.

As the porosity characteristics of cement-based concretes are largely dependent on the porosity of cement paste, it is precisely the cement paste which, as it provides the continuous phase within these composites, is in most cases responsible for ensuring the required durability of the concretes. Therefore, the purpose of this paper is to demonstrate the effect of changes in the open porosity of cement paste caused by the progressive hydration of the cement contained in the paste and its natural carbonation. These changes were subsequently compared to changes in the permeability of cement paste for gas (nitrogen) flow. Measurements of gas permeability of cement pastes have posed a considerable challenge to date due to the shrinkage cracking of specimens frequently present, especially when large specimens were used, and the paste in question exhibited a high water-cement ratio. The research results obtained in such cases differed considerably and were not entirely reliable. Therefore, the modified RILEM-Cembureau method was used in the study presented in this paper in which the permeability of cement pastes for nitrogen was determined in specimens with dimensions that were representative of this material and at the same time yielded high repeatability and homogeneity of the results obtained. This method is described in detail in Section 2.

### 1.2. Changes in Porosity Characteristics in the Context of Progressive Hydration and Carbonation of Cement Pastes

The assessment of open porosity, and in particular the impact of various environmental factors on changes of this characteristic in cement pastes has been the focus of interest of many researchers [11,12,13,14,15,16,17]. Chen et al. [18] carried out fundamental research in the field of open porosity changes in cement pastes made of ordinary Portland cement (OPC). Using the MIP method, the authors determined changes in open porosity as hydration progressed after 1, 3, 7, 14 and 28 days of cement reaction with water. The w/c ratio values adopted ranged from 0.3 to 0.7. The largest changes in open porosity (pores which could be penetrated by mercury) in the cement pastes analyzed occurred up to day seven of hydration and these ranged from around 5% to around 8%. Later, these changes were significantly smaller. On the other hand, the authors also confirm the significance of the w/c ratio for open porosity because its increase by 0.1 results in an increase in total MIP porosity by approximately 5% after 28 days of curing. The study results also indicate an interesting reduction in the amount of larger capillary pores (>100 nm) in favor of middle capillary pores (50–100 nm) and mesopores (5–50 nm), which is caused by the hydration process progressing over time; this relationship is described in quantitative terms.

There is consensus about the effect of the progress of carbonation on the reduction in open porosity and thus the transport capabilities of ordinary Portland cement pastes as well as the cementitious composites made using such cement [19,20,21,22,23]. All hardened paste ingredients are subject to carbonation irrespective of whether they have already undergone hydration or not. The most important carbonation processes of OPC cement hydration products occur according to Equations (1)–(2) [24,25,26,27]. However, owing to their volumetric proportions in the hardened paste, two of these ingredients play the most important roles from the point of view of changes in material porosity; these are CH (portlandite) and the C-S-H phase.

Ca(OH)_2_ + CO_2_ → CaCO_3_ + H_2_O (1)

3CaO·2SiO_2_·3H_2_O + 3CO_2_ + nH_2_O → 3CaCO_3_ + 2SiO_2_aq + 3H_2_O (2)

Depending on the precise polymorphic form of calcium carbonate (calcite, aragonite, vaterite), its crystallization results in a greater or lesser increase in volume relative to the solid substrates used to produce it [21].

Changes in the microstructure of hardened cement paste as a result of carbonation are explained, inter alia, in [28]. In the case of the carbonation of portlandite, this process is relatively simple and consists in the topochemical reaction of calcium hydroxide with CO_2_, with time going from the surface to the deeper layers of hexagonal crystals of Ca(OH)_2_. Depending on the ambient thermodynamic conditions, the polymorphic forms of calcium carbonate mentioned above crystallize.

Where the C-S-H phase decomposes as a result of carbonation, this process becomes more complex. According to Chen [29], the slow carbonation process leads to the decalcification of the C-S-H phase, resulting in a lower Ca/Si ratio. Initially, the degree of polymerization increases, i.e., silicon-oxygen chains become longer as the quantity of Ca^2+^ ions in the interlayer space decreases. These observations are also confirmed by Sevelsted et al. [30]. When the Ca/Si ratio reaches a value of around 1.2, this leads to a reduction in the specific surface area of the amorphous C-S-H phase. In the advanced carbonation phase when the Ca/Si ratio reaches a value of around 0.66, the complete decomposition of this phase occurs during which the cationic subnetwork consisting of an octahedral calcium-oxygen layer is destroyed and hydrated silica gel precipitates. Reactions illustrating the two-stage decomposition of the C-S-H phase were proposed by the authors of [30]; these are presented in Equations (3) and (4) where the value ranges of the x and y stoichiometric coefficients are as follows: 0 ≤ x ≤ 1.1 and 0.4 ≤ y ≤ 2.5.

(CaO)_x+0.67_·SiO_2_·(H_2_O)_y_ + xCO_2_ → (CaO)_0.67_·SiO_2_·(H_2_O)_y-z_ + xCaCO_3_ + zH_2_O (3)

(CaO)_0.67_·SiO_2_·(H_2_O)_y-z_ + 0.67CO_2_ → 0.67CaCO_3_ + SiO_2_·(H_2_O)_y-z-n_ + nH_2_O (4)

The main aim of the presented studies below was to verify the changes in permeability of cement pastes made from Ordinary Portland cement characterized by variable w/c ratios, in which during the 90 days and 2 years a natural progress of hydration and carbonation was conducted. The level of gas permeability is directly due to changes in the microstructure of the cementitious pastes, and consequently to the changes in the open porosity available for gas. Therefore, the changes in the phase composition of the pastes were determined using the X-ray diffraction (XRD) and thermogravimetry/differential thermal analysis (TG/DTA) methods and volume fraction of open porosity as well as its distribution were determined using: helium pycnometry, mercury intrusion porosimetry, and water saturation.

## 2. Experimental Procedures

### 2.1. Material and Specimen Preparation

The pastes analyzed were made of Ordinary Portland cement CEM I class 42.5 in accordance with EN 197-1 [31]. The characteristics of this cement are provided in Table 1. Cement pastes were prepared in accordance with EN 196-1 [32].

In order to minimize the possibility of paste specimens developing scratches or cracks, usually as a result of shrinkage and thermal deformations, a decision was made to minimize the dimensions of these specimens. On the basis of studies and numerous observations of our own, it was concluded that these undesirable phenomena could be avoided by using cylindrical specimens with a diameter of 10 mm and a height of around 60 mm. The dimensions of such cement paste specimens can be considered representative. The specimens were formed in rigid plastic tubes. After the tubes had been filled with paste, they were closed with appropriate plugs in order to avoid water evaporation. During the first three days of curing, the specimens thus formed were stored in a vertical position. After 28 days of curing in the absence of evaporation of the mixing water, the specimens were removed from their molds, their ends were cut (about 5 mm on each end) and they were ground to a height of 50 mm. Subsequently, the specimens were divided into two groups—the first one was designated for testing after 90 days and the second after 2 years. Specimens from the first group were cured for 28 days in insulated molds, and then for the next 62 days they were stored under laboratory conditions at a temperature of about 20 °C and relative humidity of 60 ± 5%. Before testing, the specimens were dried to constant mass at 40 °C in each case. The relatively low drying temperature adopted made it possible to limit changes in the structural characteristics of the pastes analyzed [15]. The second group of specimens was stored in the same conditions as the first one, but for two years. The two-year storage period was intended, on the one hand, to enable further progress of the cement hydration processes; on the other hand, cement paste was subject to natural carbonation as a result of being exposed to CO_2_. The vast majority of research on the carbonation of cement pastes is carried out under laboratory conditions in the chambers enabling acceleration of this process (varied parameters: temperature, humidity, CO_2_ concentration). Such conditions well reflect the progress of natural carbonation in pastes with respect to the occurring phase changes in a qualitative manner. However, the rate of carbonation processes is important not only because of the quantitative changes in the phase composition of aging cement pastes, but also their texture. This, in turn, strongly affects the other properties such as porosity and permeability. In addition, the natural long-term advancement of carbonation does not exclude simultaneous progress of hydration of clinker grains, which is practically neglected in the case of accelerated carbonation [33,34,35]. Hence, the studies were carried out under natural carbonation conditions in periods in which the changes resulting from the reaction with CO_2_ were significant.

Four w/c ratio values were adopted, which ranged from 0.3 to 0.6 with a step interval of 0.1. Such a wide range of w/c ratios is representative of cement pastes that are components of both ordinary and high-performance concretes. In order to ensure a similar liquidity of the pastes during their formation and compaction, a small amount of superplasticiser was added to the pastes with w/c ratios of 0.4 and 0.3. This procedure ensured that the mixtures in the molds were properly compacted. A slight sedimentation effect was observed in the case of pastes with a w/c ratio of 0.6. However, as already mentioned, specimen ends were ground to eliminate unrepresentative specimen portions. Due to the aforementioned tendency towards sedimentation, this treatment is particularly important in the case of pastes with a high w/c ratio.

### 2.2. Methods

#### 2.2.1. Gas Permeability

The permeability of cement pastes was determined using the RILEM-Cembureau method [36,37] for concrete and it was expressed as the coefficient of permeability. Permeability was determined for nitrogen flow.

The coefficient of permeability (k) was determined using the following equation:

(5) $$k=\;\frac{2{\mathrm{QP}}_{a}\eta L}{A\left(P^{2}-P_{a}^{2}\right)}\left(m^{2}\right)$$

where:

Q = V/t—the measured gas flow intensity (m^3^/s);
P_a_—atmospheric pressure (1 bar = 10^5^ Pa);
P—pressure (absolute) (Pa);
A—specimen cross-sectional area (m^2^);
η—viscosity of the gas; η = 17.15 (Pa·s);
L—specimen thickness (m).

**Figure 1:**
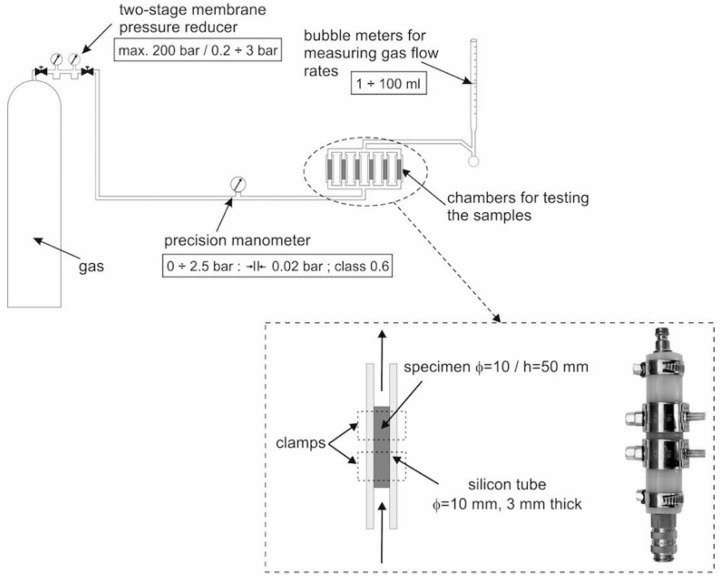
RILEM-Cembureau gas permeability measurement apparatus and details of specimen fixing in a silicon tube chamber [38].

The measurements were made using a suitably modified device which allowed the permeability of specimens with a diameter of 10 mm to be measured. The device and a detailed view of the specimen-holding chambers are schematically shown in Figure 1. The test procedure was generally in accordance with the recommendations specified in the RILEM-Cembureau method. The only deviation concerned specimen dimensions. The test method is described in detail in [38].

#### 2.2.2. Open Porosity

Open porosity in the cement pastes analyzed was determined by three methods:

- helium porosity (p_H_) calculated by comparing bulk (envelope) density with true (skeleton) density;
- open porosity determined on the basis of mercury intrusion porosimetry measurements (p_MIP_);
- open porosity determined on the basis of absorption of water (water saturation) by volume of paste specimens (p_ws_).

Helium porosity (p_H_) was calculated using the following relationship:

(6) $$p_{H}=\;\left(1-\frac{\rho_{\mathrm{bulk}}}{\rho_{\mathrm{true}}}\right)100\;\left(\%\;\mathrm{vol}.\right)$$

where:

ρ_bulk_—bulk density (g/cm^3^),
ρ_true_—true density (helium pycnometry) (g/cm^3^).

Bulk (envelope) density was determined using the GeoPyc 1360 powder pycnometer (Micromeritics, Norcross, GA, USA). The detailed procedure is described in [39]. True density was determined by helium pycnometry using the Ultrapycnometer 1200e device (Quantachrome Instruments, Boynton Beach, FL, USA). In essence, this device measures the skeleton volume of the specimen to a very high accuracy i.e., to the fourth decimal place in cm^3^. During the measurement, helium molecules fill the smallest open pores present in the material tested. According to [40], helium atoms, which are very small, may penetrate pores with a diameter as low as 0.25 nm. The detailed procedure is described in [40].

Despite its many drawbacks, like the “ink bottle” effect [41], mercury intrusion porosimetry (MIP) is still considered a very valuable method which provides a lot of information about the structure of the materials studied, including porosity characteristics which are identified within a wide range of pore diameters. On one hand, researchers often refer to these results in a qualitative manner [42], but on the other this method has been successfully used in the assessment of the microporosity structure of advanced cementitious composites such as High Performance Concretes (HPC), Ultra High Performance Concretes (UHPC) and Reactive Powder Concretes (RPC) [43,44,45] and in the assessment of microstructure changes in materials exposed to corrosive environments [25,46].

The Poremaster 60 mercury porosimeter (Quantachrome Instruments, Boynton Beach, FL, USA) with a pressure range from 0.1 to 400 N/mm^2^ was used in the tests presented here. This pressure range allowed the identification of pores with diameters ranging from 3.75 nm to approx. 0.25 mm.

The third method used in order to assess the open porosity of cement pastes was the determination of water absorption (saturation) by volume. Absorption by mass (w_a_) and bulk density (ρ_bulk_) were used in calculations of absorption by volume.

p_ws_ = w_a_ × ρ_bulk_ (7)

#### 2.2.3. Thermogravimetric (TG) Tests

TG-DTA measurements were made using the NETZSCH STA 449 F3 Jupiter device equipped with the QMS 403 Aëolos mass spectrometer (NETZSCH-Gerätebau GmbH, Selb, Germany), which enabled the analysis of the gases evolved. The tests were carried out in a range of temperatures from ambient up to 1000 °C with temperature increasing at a rate of 15 °C/min. The information obtained made it possible to determine the temperature ranges attributable to the relevant decomposition processes of the cement pastes tested:

- dehydration (Ldh), including of ettringite, monosulphate, hydrogarnets, the C-S-H phase;
- dehydroxylation (Ldx), primarily of portlandite;
- decarbonation (Ldc) primarily of calcite which appears in the paste as a result of the carbonation, mainly of portlandite but also of the C-S-H phase, hydrogarnets or ettringite.

**Figure 2:**
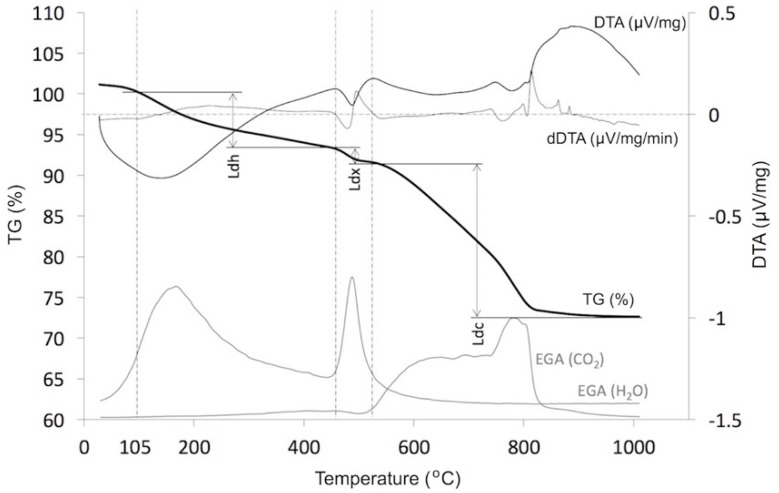
Sample TG/DTA and EGA curves as functions of temperature.

As shown in Figure 2, in order to facilitate the precise determination of the Ldx dehydroxylation range, and subsequently of the Ldh and Ldc ranges, the derivative of the DTA curve was calculated in each case so that zero dDTA values and the EGA curve of the H_2_O and CO_2_ gases released during heating made it possible to determine the start and end of the portlandite decomposition process. The temperature value at which free water was completely removed was assumed to be 105 °C. This problem is also discussed in more detail in [47].

On the basis of the test results obtained, the contents of free portlandite and of products of the carbonation process, i.e., carbonates expressed as CaCO_3_, were determined using the (8) and (9) relationship according to [48].

(8) $$\mathrm{CH}\;\left(\%\right)=\frac{M_{\mathrm{Ca}{\left(\mathrm{OH}\right)}_{2}}}{M_{H_{2}O}}\cdot \mathrm{Ldx}=4.11\cdot \mathrm{Ldx}$$

where:

Ldx—mass loss in the dehydroxylation range (%),
M_Ca(OH)_2__—molar mass of calcium hydroxide (75.09 g/mol),
M_H_2_O_—molar mass of water (18.02 g/mol).

(9) $$\mathrm{Carbonates}\;\left(\%\right)=\frac{M_{{\mathrm{CaCO}}_{3}}}{M_{{\mathrm{CO}}_{2}}}\cdot \mathrm{Ldc}=2.27\cdot \mathrm{Ldc}$$

where:

Ldc—mass loss in the decarbonation range (%),
M_CaCO_3__—molar mass of calcium carbonate (100.09 g/mol),
M_CO_2__—molar mass of carbon dioxide (44.01 g/mol).

Additionally, using Bhatty’s [49] method, the degree of cement hydration (α) was determined by calculating the amount of chemically bound water W_B_ according to Equation (10):

(10) $$\alpha =\frac{W_{B}}{W_{B\infty }}\cdot 100$$

(11) $$W_{B}=\mathrm{Ldh}+\mathrm{Ldx}+0.41\cdot \mathrm{Ldc}$$

where:

W_B_—the amount of chemically bound water after time t [%],
W_B__∞_—the amount of chemically bound water after hydration has been completed [%].

According to Bhatty [49], the denominator in relationship (10) has a constant value of 0.24. The issue of the amount of chemically bound water at full Portland cement hydration has often been discussed and the W_B__∞_ value has a fairly wide range from 0.20 to 0.23 [50,51,52]. Additionally, the temperature at which the hydration process takes place also affect the amount of chemically bound water. This problem was addressed by [53], and therefore in order to estimate the degree of cement hydration value W_B__∞_ = 0.22 was adopted. The value of the coefficient for Ldc in Equation (11), which is 0.41, follows from the molar mass of thermally removed CO_2_ relative to the molar mass of H_2_O (crystallization water) in CH.

In the XRD analysis, the whole volume of hardened cement pastes was powdered and subjected to the tests. The XRD patterns of cement pastes were collected using an X-ray Philips/PANalytical X’Pert Pro diffractometer equipped with software containing Database ICDD PDF4+ (Malvern Panalytical Ltd, Malvern, UK).

## 3. Porosity and Permeability Test Results

The results obtained in tests of true density, bulk density and open porosity calculated on the basis of comparison between these density values and on the basis of porosimetric measurements and water saturation are presented in Table 2. The Table also includes the results of gas permeability tests. The results presented are in each case the average value of three measurements and refer to tests conducted both after 90 days and after 2 years of curing.

The test results obtained exhibited considerable homogeneity, which was adequate to the characteristic tested in each case. Further results are presented in the form of charts and a brief description of the changes observed is provided.

### 3.1. Bulk (Envelope) Density and True Density Versus w/c Ratio

**Figure 3:**
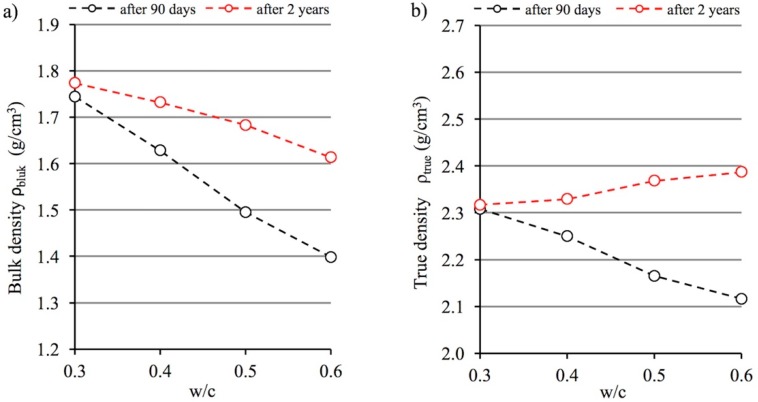
Relationship between bulk (**a**) and true (**b**) density versus the w/c ratio of cement pastes after 90 days and 2 years of curing.

The test results shown in Figure 3a clearly indicate that the w/c ratio has a very significant effect on the bulk density value. As the w/c ratio increases, density decreases both after 90 days and after 2 years of curing. The decrease in density is significant because when the densities of pastes made using extreme w/c values are compared, it amounts to around 20% after 90 days of curing. After a longer period, this decrease is smaller by half and amounts to around 10%. The variation in density depending on curing time is a very interesting phenomenon. A longer curing period of two years is associated with higher cement hydration levels, but also the development of carbonation processes (see Section 4, Table 3). These interactions result in, *inter alia*, an increase in the bulk density of cement pastes after 2 years compared to the density of pastes after 90 days. Most interestingly, differences in these values rise together with the w/c ratio. Bulk density after two years increased by 1.7% at a w/c ratio of 0.3, while for a paste with a w/c ratio of 0.6 this increase was as much as 15.4%.

True density, which is the density of the material skeleton, is a characteristic independent of material porosity, which assumes that only open pores are present in the material structure. In fact, it is precisely this type of porosity that is observed in cement pastes. Thus, skeletal density depends on the density of the products resulting from cement hydration, the occurrence of carbonation processes and the presence of relict clinker grains. It was observed that after 90 days of curing of cement paste, its true density decreases significantly as the w/c ratio increases. This relationship appears to be obvious due to the fact that at higher w/c ratios, the cement paste includes a greater amount of lower-density hydration products in its structure. As the w/c ratio increases, a higher hydration level is observed (see Table 3), and therefore there is a lower content of non-hydrated cement grains with greater density. However, the 2-year storage period of cement pastes under laboratory conditions reversed the relationship observed after 90 days. After 2 years, true density increases together with the increase in the w/c ratio, with the changes observed being primarily driven by the carbonation process. The difference in true density after two years and 90 days increases together with the w/c ratio of the paste series analyzed; i.e., together with the potential for carbonation. For higher w/c ratios, cement paste microstructure is characterized by higher porosity, especially capillary porosity, and a higher proportion of the (CH) phase, which is subject to rapid carbonation. 

### 3.2. Open Porosity and w/c Ratio

**Figure 4:**
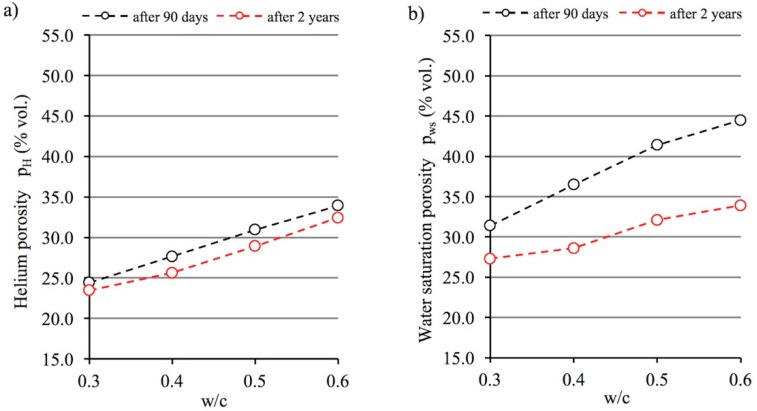
Relationship between helium (**a**) and water saturation (**b**) porosity versus the w/c ratio of cement pastes after 90 days and 2 years of curing.

In Figure 4a below, open porosity determined by two methods is compared depending on the w/c ratio. Open porosity, also called helium porosity, was calculated according to Formula (6) described in Section 2.2.2. It is clear that open porosity strongly depends on the w/c ratio of cement paste. A generally known trend is observed: as the quantity of water increases and the amount of cement decreases, open porosity increases. This relationship is a quasi-linear one. A two-year curing period resulted in a decrease in helium porosity in all the cement paste series analyzed. Porosity still increases together with the water-cement ratio, but porosity values are slightly lower on average about 1.6%.

Figure 4b shows open porosity results determined on the basis of the water saturation of paste specimens for different w/c ratios and curing periods. In a similar manner to the relationship described above regarding helium porosity, water saturation porosity depends very significantly on the w/c ratio. The higher the ratio, the greater the volume of water absorbed during paste saturation tests. However, if we compare water saturation porosity and helium porosity values after 90 days of curing pastes with the same w/c ratios it can be observed that the water saturation porosity value is clearly greater. Reasons for this difference are explained in [38]. Water saturation porosity does not reflect the actual open porosity of materials, since water, being highly polar, is adsorbed by the gel formed by hydrated calcium silicates. Water molecules fill interlayer spaces, thus increasing the distances between them and creating “additional porosity”. This effect was observed by, among others, Krus et al. [12] who described the swelling of cement binders stored in water.

The two-year curing period, which resulted in further progress in cement hydration and carbonation, contributed to a large reduction in water saturation porosity. These processes caused a drop in the porosity assessed in this manner by as much as 10.6% (from 44.5% to 33.9%) by volume in cement paste with a w/c ratio of 0.6. Figure 4 shows that the reduction in porosity depends on the w/c ratio. The higher the w/c ratio, the higher the porosity, especially capillary porosity. Also, the higher the potential for carbonation, the greater the decrease in water saturation porosity. It should be noted that after two years of curing, water saturation porosity is still higher than helium porosity, but the difference drops significantly—by half on average. This may prove that the further hydration of cement, in combination with carbonation, results in a decrease in the amount of C-S-H gel which is susceptible to an increase in interlayer space as a result of such space being filled with water. In other words, in addition to the slow progress of relict cement grain hydration, a complete destruction of C-S-H phase chains as described by Equation (4) may be taking place.

**Figure 5:**
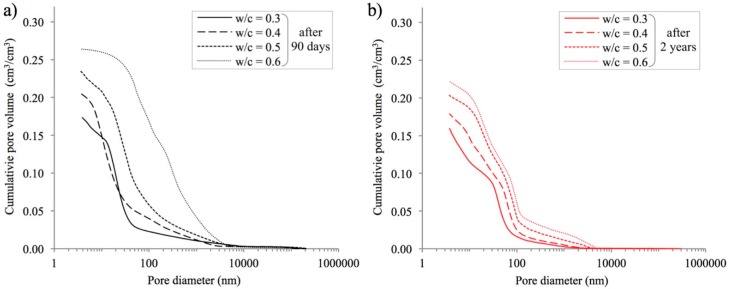
Cumulative pore distribution curves in cement pastes with different w/c ratios after 90 days (**a**) and 2 years of curing (**b**).

A comparison of not just total porosity, but also of pore distribution characteristics, is of key importance for the relationships observed. The open porosity characteristics of the cement pastes analyzed were determined on the basis of the mercury intrusion porosimetry method. This method allowed for the quantitative identification of pores with diameters ranging from 3.75 nm to approximately 0.25 mm. Cumulative pore distribution curves are presented in Figure 5 below.

When analyzing the distributions presented, several patterns can be discerned. Firstly, after 90 days of curing, total porosity as determined by the mercury intrusion porosimetry method strongly depends on the w/c ratio. As in the case of porosity assessed using the methods previously described, porosity increases together with the increase in this ratio. Secondly, in addition to differences in total porosity, there are changes in the proportions of pores with different diameters. Therefore, the nature of the cumulative curves presented changes depending on the value of the w/c ratio. Moreover, after two years of curing and therefore also of carbonation, porosity decreases. The decrease in porosity is greater the higher is the w/c ratio. In the case of w/c 0.3 decrease was 1.4%, while for w/c 0.6 was three times greater.

**Figure 6:**
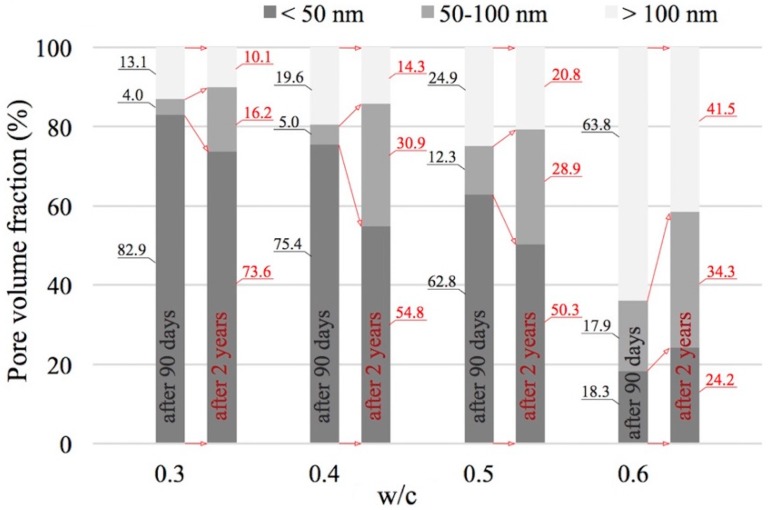
Proportions of different pore classes in pastes with different w/c ratios after 90 days and 2 years of curing.

In order to obtain a better description of the changes observed in porosity characteristics, especially those caused by longer curing periods and carbonation, a quantitative assessment of the groups of pores distinguished according to [18,54] was conducted. The entire range of pores identified by mercury intrusion porosimetry was divided into three classes: mesopores (<50 nm), middle capillary pores (50–100 nm), and larger capillary pores (>100 nm). The results of this analysis are shown in Figure 6.

The above analysis confirms earlier observations regarding cumulative distribution curves (Figure 5): an increase in the w/c ratio results in an increase in the proportion of capillary porosity. For example, in the paste with a w/c ratio of 0.3, capillary pores (>50 nm) accounted for 17.1% of the total, whereas in the paste with a w/c ratio of 0.6, their proportion increased to 81.7%. Obviously, as the capillary pore proportion increases, the proportion of mesopores, i.e., pores with a diameter below 50 nm, decreases. Very interesting changes in the proportions of pores belonging to individual classes can be observed when comparing the numbers after 90 days and 2 years. Firstly, from the results shown in Figure 6, we can conclude that the two-year curing period caused a significant reduction in the proportion of larger capillary pores (>100 nm). In the case of paste with a w/c ratio of 0.3, the decrease is from 13.1% to 10.1%, and for a w/c ratio of 0.6 the initial value of 63.8% drops to 41.5%. The decrease in the proportion of larger capillary pores (>100 nm) is accompanied by an increase in the proportion of medium capillary pores; i.e., pores with diameters ranging from 50 to 100 nm. In each of the series of paste specimens analyzed, on average a more than twofold increase in this respect was observed. As concerns changes in the proportion of mesopores, the differences are not large. Generally, it can be concluded that at low w/c ratios, the proportion of pores with diameters <50 nm slightly decreases and at high w/c ratios, it slightly increases.

The observations described above clearly indicate that open porosity, and pore distribution characteristics in particular, depends on w/c ratios but also on curing time, and thus the progress of the hydration and carbonation processes.

### 3.3. Gas Permeability and w/c Ratio

The permeability determined for the flow of any medium present in the operating environment of cementitious composites provides valuable information about the accessibility of their porous structure to this medium. Studies based on the assessment of gas flow intensity make it possible to arrive at particularly subtle distinctions related to the material’s internal accessibility. Nitrogen molecules are able to penetrate open pores with very small diameters, even below 1 nm [55]. The main purpose of the test results presented was to determine how the changes in porosity characteristics of cement pastes described above affect their permeability determined by nitrogen flow.

**Figure 7:**
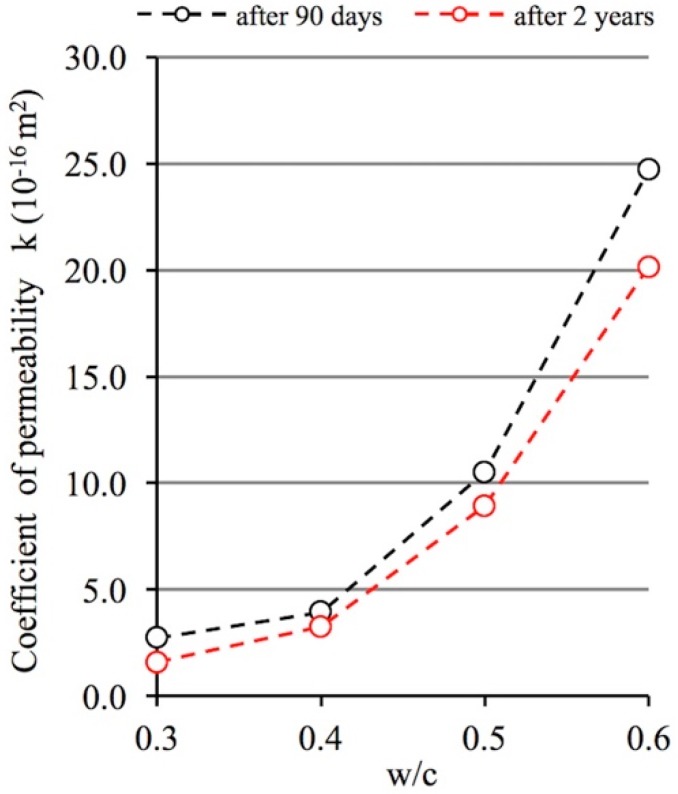
Relationship between permeability and the w/c ratio of cement pastes after 90 days and 2 years of curing.

In Figure 7 below, the results included in Table 2 concerning the effect of the w/c ratio and curing time on the permeability of cement pastes are presented.

Similarly to open porosity, permeability—both after 90 days and two years of curing—strongly depends on the w/c ratio. In [38], solely on the basis of the results obtained after 90 days of curing, the relationship between permeability and the w/c ratio was determined by means of exponential regression equations and high determination coefficients (R^2^ > 0.93) were obtained. Two-year hydration period combined with the carbonation process causes significant changes in the porosity of the pastes analyzed and therefore, significant changes in their permeability should also be expected. Indeed, permeability tests carried out after 2 years of curing show values lower than those recorded after 90 days. The average decrease in permeability after 2 years compared to the permeability after 90 days is around 23%, and the range is from 15% to 42%. It can also be observed that in general, the greatest drops in permeability were recorded for paste with w/c ratio of 0.3. It appears, therefore, that the reason for the phenomenon observed is the fact that gas penetrates not only the pores identified using the mercury intrusion porosimetry method, but also pores with even smaller diameters. Thus, further hydration and carbonation result in a significant decrease in the permeability of pastes characterized by high proportions of mesopores.

## 4. Results of Tests on the Degree of Hydration and Carbonation 

### 4.1. XRD Test Results

Only cement pastes, which were characterized by extreme w/c ratio values, i.e., 0.3 and 0.6, were subjected to X-ray tests. The tests were carried out for paste specimens after 90 days and after 2 years of curing.

**Figure 8:**
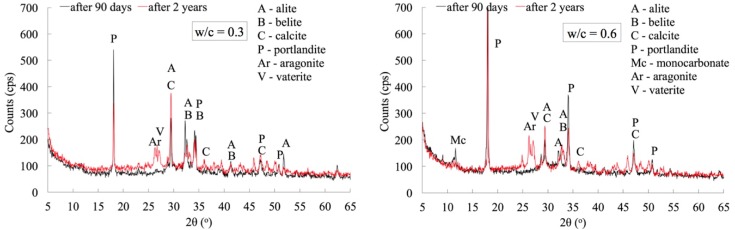
X-ray pattern of cement pastes with w/c ratios of 0.3 and 0.6 after 90 days and 2 years of curing.

The X-ray patterns shown in Figure 8 clearly indicate the progress of the carbonation and hydration processes between 90 days and 2 years of curing under laboratory conditions. In all cases of the studied materials, the presence of the basic phases naturally occurring in the incompletely carbonated cement pastes, i.e., calcite and portlandite, was found. In the case of pastes with w/c = 0.3, the peak assigned to the alite (2θ = 51°) after 90 days of hydration indicate the presence of relicts of cement grains gradually disappearing with the progress of time and thus with the increase of hydration degree. This process is reflected in the X-ray pattern carried out after 2 years. In addition, in the case of paste with w/c of 0.6, significantly higher hydration degree caused the disappearance of the characteristic peak ascribed to alite at 2θ = 51°. Moreover, regardless of the water-cement ratio after a longer, 2-year period of time, a peak characteristic for calcite 2θ = 36° appears. Previously, after 90 days of simultaneous progress of hydration and carbonation, this peak was not detected.

A common feature of the cement pastes analyzed, regardless of the water-cement ratio, is the crystallization of the remaining polymorphic forms of calcium carbonate, i.e., vaterite and aragonite. However, these phases are only formed after a longer period of natural carbonation, because their characteristic peaks are observed only after 2 years of maturation. In the case of cement paste with w/c = 0.6, crystallization of monocarbonate C_3_A·CaCO_3_·11H_2_O after 90 days of maturation was also observed, which may be the result of the progress of carbonation of both hydrated calcium aluminates [25] and sulphoaluminate phases [26]. In a longer period of time; i.e., after 2 years, this phase disappears.

### 4.2. TG/DTA Test Results

**Figure 9:**
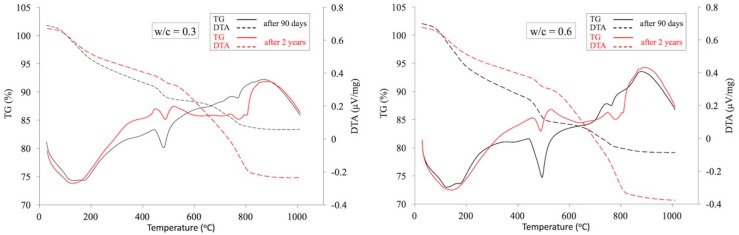
TG/DTA analyses of pastes with w/c ratios of 0.3 and 0.6 after 90 days and 2 years of curing.

In a similar manner as in the case of XRD tests, analogous cement paste specimens made with water-cement ratios of 0.3 and 0.6 were subject to thermogravimetric analysis. TG/DTA analyses of cement pastes are summarized in Figure 9 below. Table 3 presents their dehydration (Ldh), dehydroxylation (Ldx), and decarbonation (Ldc) ranges together with the temperature ranges determined. The values obtained for these parameters correspond well with those described in the literature [47]. On this basis, the quantities of CH portlandite and carbonates expressed as CaCO_3_ as well as the amount of chemically bound water and the degree of cement hydration were calculated using Equations (8)–(11).

The results obtained clearly illustrate the positive effect of both curing time (cement paste age) and the amount of mixing water on the degree of cement hydration. The highest degree of hydration was exhibited by cement paste, reaching a value of 86% after 2 years of curing for a w/c ratio of 0.6. The lowest degree of hydration, i.e., around 62% for the 90-day curing period and amount of mixing w/c = 0.3.

The reduction in the amount of CH caused by the progress of carbonation between 90 days and 2 years of curing was around 30% in the case of the w/c ratio of 0.3. While it increased to more than 50% when more mixing water was used. The amount of precipitated portlandite after 90 days of curing was also clearly related to the amount of mixing water. When comparing pastes with w/c ratios of 0.3 and 0.6, this amount approximately doubles. 

The progress of paste carbonation is clearly observed irrespective of the w/c ratio. However, as should have been expected, this phenomenon is much more visible at the high w/c ratio of 0.6. This is mentioned, *inter alia*, by Wang and Czarnecki [56,57]. The amount of carbonates expressed as CaCO_3_ increases 3.7 times after 2 years of curing. In the case of the w/c ratio of 0.3 after 2 years this value is 3.1 times higher. 

## 5. Discussion of Study Results

The qualitative changes occurring with respect to true density and bulk density, and thus the porosity of the pastes analyzed can be explained in terms of the density values of the individual ingredients of hardened and carbonated cement paste presented in Table 4 [21,58]. The table presents the results of calculations concerning the volume changes of solid products as compared to substrates. In the case of portlandite carbonation, calculations were conducted for the three possible polymorphic forms of CaCO_3_, i.e., calcite, aragonite and vaterite, which crystallize within pastes. Additionally, in the case of C-S-H phase carbonation, calculations were conducted for the process stoichiometry stated in Equation (2), assuming the three possible forms of calcium carbonate just as for CH.

In each case of cement paste the emergence of the hydration products of the binders analyzed is associated with a decrease in their true density (see Table 1 and Table 4). Therefore, the true density observed after 90 days decreases as the w/c ratio increases, and thus also as the degree of hydration increases (see Table 3). On the other hand, after a longer, 2-year curing period under natural carbonation conditions, this density increases and the increase is higher where the value of the w/c ratio is higher; i.e., the paste’s potential for carbonation is greater. The highest true density values were obtained for pastes with a w/c ratio of 0.6, which is associated—as thermogravimetric studies confirm—with the highest degree of carbonation. When the true density values contained in Table 4 have been analyzed, the changes observed appear fully justified, since all carbonation products, irrespective of the particular polymorphic form of calcium carbonate, exhibit a higher true density compared to both portlandite and the C-S-H phase.

Changes in the volume of substrates compared to products as a result of portlandite carbonation can be calculated relatively easily, as shown in Table 4. The smallest increase in volume can be expected during crystallization of aragonite, and the greatest increase where vaterite is formed. In the case of C-S-H phase carbonation, the changes in its volume are difficult to predict and depend on multiple factors, i.e., the Ca/Si ratio, the amount of water bound within the phase, the amount of water bound within the silica gel and the specific polymorphic form of the precipitating calcium carbonate. However, according to Morandeau et al. [59] C-S-H carbonation resulting in the crystallization of calcite leads to a decrease in porosity, which is consistent with the results presented above. Moreover, assuming that the carbonation of the C-S-H phase progresses according to the stoichiometric relationship given in Equation (2) and assuming that the water bound within the silica gel has been completely removed as a result of the material drying and leaving amorphous anhydrous silica with a density of 2.20 g/cm^3^, and also assuming the most unfavorable crystallization variant for calcium carbonate, i.e., aragonite, it can be calculated that product molar volumes increase by around 15% as compared to substrate molar volumes (see Table 4). In other words, when considering other possible CaCO_3_ forms, greater increases in the volume of products relative to substrates can be expected.

Summing up, the carbonation of both CH and C-S-H generally involves an increase in the volume of products as a result of reactions with CO_2_, which leads to a drop in the porosity of cement pastes.

As has been mentioned before, the main purpose of the studies and analyses conducted was, on the one hand, to quantify changes in permeability in cement pastes with different w/c ratios as a result of the longer curing period which involves the effects (processes) described. On the other hand, however, the focus was on characterizing changes in the porosity structure of the pastes analyzed and the impact of these changes on their permeability.

Gas permeability, which is a measure of the accessibility of a porous structure to such a medium, exhibits very large variations which depends on the w/c ratio. The impact of this factor can be successfully described using exponential functions, as demonstrated in [38]. In spite of this, the author tends to agree with Hamami [60], among other authors, who states that knowledge of total porosity is not always sufficient to predict permeability. As demonstrated by Garboczi [61] and also by Katz and Thompson [62], permeability is a function both of the total amount of open pores and of their distribution.

The cement pastes analyzed were exposed for two years to laboratory conditions which caused significant changes in the porosity structure of these pastes and contributed to the reduction of permeability. The qualitative changes which occurred could be predicted, but in quantitative terms, greater differences between these values were expected.

The average decrease in permeability after 2 years compared to permeability after 90 days was around 23%. However, the greatest drop 42% was registered in the case of w/c ratio of 0.3. The reason for the phenomenon observed is the fact that further progress of hydration and carbonation occurring in tight pastes that exhibit low open porosity (with a dominant pore diameter <50 nm) has the greatest impact on the decrease in permeability in these pastes.

## 6. Conclusions

The research results presented as well as the analyses carried out concerning changes in open porosity characteristics and the permeability of cement pastes make it possible to formulate the following conclusions and observations.

Gas permeability, which is a characteristic resulting directly from the open porosity characteristics of the pastes analyzed, exhibits considerable differences in the values recorded depending on the w/c ratio irrespective of the length of the curing period. The two-year curing period and the accompanying hydration and, above all, carbonation processes led to a decrease in permeability in each cement paste series analyzed. The decrease ranged from 15% to 42% depending on the w/c ratio. It was also found that for a low w/c ratio, i.e., in pastes characterized by high tightness and low open porosity with a high proportion of mesopores, the reduction in permeability was the highest.

Two years of curing under laboratory conditions resulted in further cement hydration, but primarily contributed to the carbonation of cement paste ingredients. The level of carbonation expressed as the amount of calcium carbonates depended on the w/c ratio of the paste.

The carbonation of both CH and C-S-H as a result of reactions with CO_2_ causes an increase in the volume of products relative to the volume of substrates, which results not only in lowering total open porosity, but primarily in a change in porosity distribution. Carbonation causes a significant reduction in the proportion of larger capillary pores (>100 nm). The extent of this change is greater where more capillaries are present in the structure of the paste analyzed. Thus, the reduction of the proportion of large capillary pores becomes more pronounced together with an increase in the w/c ratio and together with an increase in the availability of CO_2_ to substrates which can react with it, forming carbonation products. The decrease in the proportion of larger capillary pores (>100 nm) in the range analyzed is accompanied by, on average, a more than doubling of the proportion of medium capillary pores, i.e., pores with diameters ranging from 50 to 100 nm.

## Figures and Tables

**Table 1:** Chemical and physical characteristics of cement CEM I 42.5 R.

**Chemical characteristics (oxide analysis, % by mass)**											
SiO_2_	Al_2_O_3_	Fe_2_O_3_	CaO	MgO	SO_3_	Na_2_O		K_2_O	eqNa_2_O	Cl^−^	Portland clinker content
18.6	5.3	2.9	62.7	1.50	3.22	0.19		0.96	0.82	0.060	96
**Physical characteristics**											
Specific area (Blaine method), m^2^/kg			True density, g/cm^3^		Setting time (minutes)				Compressive strength, MPa		
					initial		final		after 2 days		after 28 days
340			3.09		199		270		29.3		55.1

**Table 2:** Results of density, porosity and permeability tests for the cement pastes tested.

w/c Ratio	Properties					
	Bulk Density ρ_bulk_ (g/cm^3^)	True Density ρ_true_ (g/cm^3^)	Helium Porosity p_H_ (% vol.)	MIP Porosity p_MIP_ (% vol.)	Water Saturation Porosity p_WS_ (% vol.)	Coefficient of Permeability k (10^−16^ m^2^)
after 90 days						
0.3	1.744	2.308	24.4	17.3	31.4	2.73
0.4	1.628	2.250	27.6	20.5	36.5	3.95
0.5	1.495	2.165	30.9	23.5	41.4	10.50
0.6	1.398	2.116	33.9	26.5	44.5	24.70
after 2 years						
0.3	1.773	2.317	23.5	16.0	27.3	1.58
0.4	1.732	2.329	25.6	17.9	28.6	3.25
0.5	1.683	2.368	28.9	20.4	32.1	8.93
0.6	1.613	2.387	32.4	22.2	33.9	20.15

**Table 3:** Results of the tests conducted.

w/c Ratio	Ldh (%)	Ldx (%)	Ldc (%)	Ca(OH)_2_ (%)	CaCO_3_ (%)	α (%)
	after 90 days					
0.3	9.04	2.37	5.29	9.7	12.0	61.7
0.6	11.32	4.17	5.35	17.1	12.1	80.4
	after 2 years					
0.3	7.16	1.60	16.44	6.6	37.3	70.5
0.6	6.74	1.70	18.93	7.7	44.8	85.7

**Table 4:** Changes in the volumes of substrates and products as a result of carbonation processes.

	Solid substrates	→	Solid products	
	Portlandite	→	Calcite	
Molar mass (g/mol)	74.08		100.09	
True density (g/cm^3^)	2.23		2.71	
Molar volume (cm^3^/mol)	33.22		36.93	
V_products_/V_substrates_	1.11			
	Portlandite	→	Aragonite	
Molar mass (g/mol)	74.08		100.09	
True density (g/cm^3^)	2.23		2.93	
Molar volume (cm^3^/mol)	33.22		34.16	
V_products_/V_substrates_	1.03			
	Portlandite	→	Vaterite	
Molar mass (g/mol)	74.08		100.09	
True density (g/cm^3^)	2.23		2.54	
Molar volume (cm^3^/mol)	33.22		39.41	
V_products_/V_substrates_	1.19			
	C_3_S_2_H_3_ phase	→	Calcite	Amorph. silica
Molar mass (g/mol)	342.4		100.09	60.08
True density (g/cm^3^)	2.50		2.71	2.20
Molar volume (cm^3^/mol)	136.96		36.93	27.31
V_products_/V_substrates_	1.21			
	C_3_S_2_H_3_ phase	→	Aragonite	Amorph. silica
Molar mass (g/mol)	342.4		100.09	60.08
True density (g/cm^3^)	2.50		2.93	2.20
Molar volume (cm^3^/mol)	136.96		34.16	27.31
V_products_/V_substrates_	1.15			
	C_3_S_2_H_3_ phase	→	Vaterite	Amorph. silica
Molar mass (g/mol)	342.4		100.09	60.08
True density (g/cm^3^)	2.50		2.54	2.20
Molar volume (cm^3^/mol)	136.96		39.41	27.31
V_products_/V_substrates_	1.26			
